# The impact of workaholism on the general health and family relationships of nurses

**DOI:** 10.1192/j.eurpsy.2026.12046

**Published:** 2026-07-31

**Authors:** A. M. Cybulska, E. Nowrot, E. Grochans, D. Schneider-Matyka, M. Nowak, K. Rachubińska

**Affiliations:** 1 Department of Nursing; 2 Science Club at Department of Nursing, Pomeranian Medical University in Szczecin, Szczecin, Poland

## Abstract

**Introduction:**

Workaholism, defined as a compulsive and excessive involvement in professional duties to the detriment of personal life, has been identified as a significant risk factor for impaired family functioning among nurses. Prolonged working hours, heightened occupational stress, and the emotional demands inherent to nursing practice contribute to the weakening of family bonds, increased conflict, and social isolation.

**Objectives:**

The primary objective of this investigation was to evaluate the impact of workaholism on family relationships among nurses and to determine the extent to which excessive professional commitment shapes family dynamics and psychosocial wellbeing.

**Methods:**

A cross-sectional study was conducted among 165 Polish nurses. Data were collected using a diagnostic survey method, which included both a researcher-designed questionnaire and validated instruments: the Dutch Work Addiction Scale (DUWAS), the Utrecht Work Engagement Scale (UWES-17), the Survey Work–Home Interaction Nijmegen (SWING), and the General Health Questionnaire (GHQ-28).

**Results:**

Overall levels of workaholism were moderate, with a predominance of excessive work investment. Elevated workaholism scores were most frequently observed among nurses with higher education, those employed on civil contracts, working rotating shifts, holding multiple jobs, and employed in surgical wards. Work engagement was moderately high, particularly in the domain of dedication, with the highest levels observed among permanently employed nurses working in a single institution. Negative work–family interactions were most prevalent among shift workers, individuals performing 12-hour shifts, and those employed in multiple facilities. Workaholism was associated with both negative and positive spillover effects between work and private life. Poorer mental health outcomes (GHQ-28) were reported by nurses on contracts, night-shift workers, and those holding multiple jobs, with significant associations found with somatization, insomnia, depression, and social dysfunction.

**Conclusions:**

The findings indicate that the intensity and structural organization of nursing work exert a substantial influence on both mental health and the work–life balance of nurses. A moderate yet significant level of workaholism, in conjunction with average levels of work engagement, was strongly associated with occupational strain and employment conditions. These results underscore the necessity of implementing organizational interventions aimed at mitigating workaholism and promoting the psychosocial wellbeing of nurses, thereby supporting both professional performance and family functioning.

**Disclosure of Interest:**

None Declared

